# Evolved polymerases facilitate selection of fully 2′-OMe-modified aptamers†
†Electronic supplementary information (ESI) available: Methods, supplementary scheme, tables and figures. See DOI: 10.1039/c7sc03747c


**DOI:** 10.1039/c7sc03747c

**Published:** 2017-10-16

**Authors:** Zhixia Liu, Tingjian Chen, Floyd E. Romesberg

**Affiliations:** a Department of Chemistry , The Scripps Research Institute , 10550 North Torrey Pines Road, La Jolla , CA 92037 , USA . Email: floyd@scripps.edu

## Abstract

RNA or DNA aptamers with 2′-OMe-modifications have been pursued to increase resistance to nucleases, but have been difficult to identify because the OMe groups ablate polymerase recognition. We recently reported evolution of the thermostable DNA polymerases SFM4-6 and SFM4-9, which enable the efficient “transcription” and “reverse transcription”, respectively, of 2′-OMe oligonucleotides. With these polymerases, we now report the first selection of fully 2′-OMe modified aptamers, specifically aptamers that bind human neutrophil elastase (HNE). Two aptamers, 2mHNE-1 and 2mHNE-2, were isolated after five rounds of selection, and four more, 2mHNE-3–6, after an additional five rounds that included selection pressure for binding in the presence of serum. All six aptamers bind with reasonable affinity, which requires the 2′-OMe substituents. Further characterization of one aptamer, 2mHNE-5, showed that unlike a previously reported natural anti-HNE aptamer, affinity for HNE is retained in the presence of high concentrations of salt or serum. The polymerases SFM4-6 and SFM4-9 should prove valuable for the production and further exploration of modified aptamers.

## Abstract 2

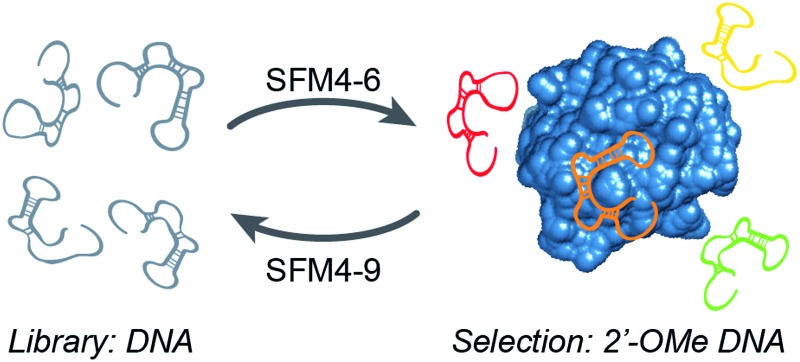
Evolved DNA polymerases are used in selections with fully 2′-OMe modified libraries to identify aptamers with high affinity for HNE.

## Introduction

Aptamers, RNA or single-stranded DNA (ssDNA) oligonucleotides that bind to a specific target, are useful affinity reagents and potentially promising therapeutics.^1^–^3^ Aptamers are discovered by creating pools of oligonucleotides with randomized sequences, enriching the pools for members that bind to a desired target (*e.g.* selection), and then amplifying the enriched pools and subjecting them to additional rounds of selection. While both DNA and RNA aptamers may be developed relatively quickly and inexpensively, their use is limited by the inherent instability of natural oligonucleotides in biological solutions due to nuclease degradation.^4^,^5^ Thus, there is significant interest in the discovery of modified aptamers that are more resistant to nucleases, with 2′-OMe and 2′-F substituents having received the most attention. In particular, 2′-OMe substituents are desirable because they impart the highest level of nuclease resistance and are also relatively inexpensive as triphosphates. Moreover, while the discovery of aptamers with high affinity to desired targets is now routine, a second limitation in aptamer development is the identification of aptamers with high specificity, which is generally more challenging. This is particularly true with positively charged protein targets due to the dominance of non-specific electrostatic interactions with the negatively charged phosphate backbone.^6^ Modifications such as 2′-OMe substituents could potentially facilitate the formation of, or even directly engage in, more specific interactions, but this has not been extensively investigated.

Aptamers with 2′-modifications have historically been generated *via* post-selection modification of a natural aptamer,^7^,^8^ but their introduction often interferes with the selected activity. The straightforward inclusion of 2′-modifications in the selection process itself is challenging, because they generally interfere with polymerase recognition. While combinations of polymerases, polymerase mutants, and/or specific reaction conditions have been identified that allow for the inclusion of various types of modified sugars,^4^,^9^,^10^ the inclusion of 2′-OMe modifications has proven particularly challenging. The only known, successful example using 2′-OMe modifications in SELEX was provided by Burmeister *et al.*^11^ who reported that with precisely optimized conditions, the Y639F/H784A/K378R triple mutant of T7 RNA polymerase can transcribe DNA into nearly fully 2′-OMe modified oligonucleotides, which along with reverse transcription by Thermoscript reverse transcriptase and amplification of the natural DNA, allowed selection of modified aptamers that bind vascular endothelial growth factor (VEGF). However, chemical synthesis was used to supplement the transcribed library for selection and the transcription reactions required the addition of unmodified GTP, indicating that at least the majority of sequences could not be transcribed in their fully modified form. Reports of additional T7 RNAP mutants with improved recognition of 2′-OMe substrates^12^,^13^ are notable, but no report of their use to select for modified aptamers has yet been reported.

To enable the more facile discovery of 2′-modified aptamers, we recently reported the directed evolution of variants of the Stoffel fragment of Taq DNA polymerase that better recognize 2′-modified substrates.^14^ One variant, SFM4-3, was found to efficiently and directly PCR amplify 2′-F-modified oligonucleotides,^14^ and we used it to evolve 2′-F-purine modified aptamers that bind human neutrophil elastase (HNE),^15^ a serine protease associated with numerous inflammatory diseases.^16^,^17^ Interestingly, HNE is positively charged, and the 2′-F substituents were found to decrease non-specific electrostatic interactions mediated by the negatively charged oligonucleotides in favor of a more specific mode of molecular recognition. In addition, we evolved two polymerase variants, SFM4-6 and SFM4-9, that more efficiently “transcribe” and “reverse transcribe” fully 2′-OMe modified oligonucleotides, respectively.^14^ Here, we apply these evolved polymerases to selections for HNE binders using libraries of fully 2′-OMe modified sequences, identifying several with high affinity, specificity, and stability against nuclease degradation.

## Results and discussion

**Fig. 1:**
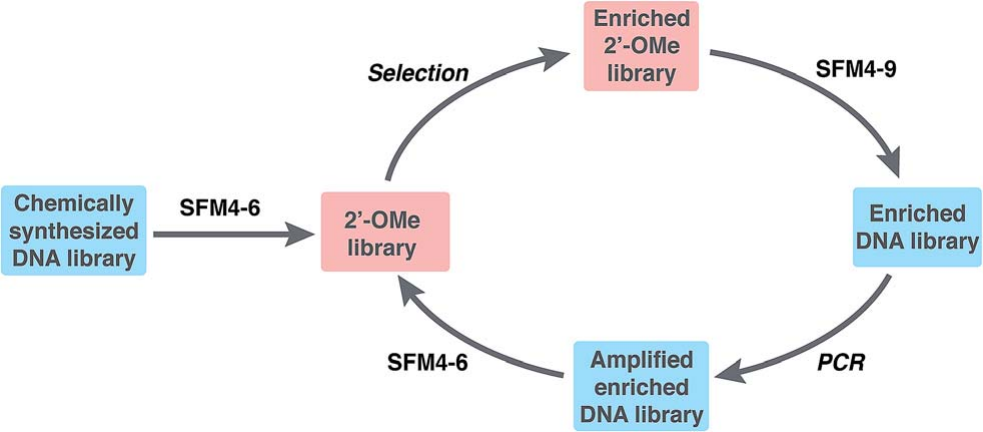
Library preparation and selection scheme.

To explore the use of our evolved polymerases for the more facile discovery of 2′-OMe-modified aptamers, we initiated selections for fully modified HNE binders using SFM4-6 and SFM4-9. A library of ∼3 × 10^14^ 67-mer ssDNA fragments with a central 30-mer random region flanked by primer binding sequences was first transcribed into a 2′-OMe-modified library using SFM4-6 and a fully 2′-OMe-modified primer (Fig. 1 and Scheme S1†). The transcription product was incubated with TurboDNase to generate a DNA-free, single-stranded 2′-OMe oligonucleotide library of ∼6 × 10^13^ members, which was heated to 95 °C and then cooled slowly to allow for secondary structure formation. The library was then subjected to selection for HNE binding (Fig. 1). Briefly, the library was incubated with immobilized HNE, subjected to washing with binding buffer (20 mM HEPES, pH 7.5, 150 mM NaCl, 6 mM KCl, 2 mM MgCl_2_), and the enriched library was recovered by eluting with hot formamide. Using SFM4-9, the recovered oligonucleotides were then reverse transcribed into DNA and PCR amplified with OneTaq DNA Polymerase (New England Biolabs). The process of transcription, selection, reverse transcription, and amplification was then repeated with the selection stringency progressively increased each round by including a negative selection step consisting of pre-incubation in an empty well and then in a well coated with bovine serum albumin, in addition to employing longer wash times and adding increasing amounts of yeast tRNA to the binding buffer (up to 3000 μg mL^–1^), to compete with non-specific 2′-OMe oligonucleotide binding (Scheme S1 and Table S1†).

After five rounds of selection, we sequenced 25 clones and found two that were enriched, 2mHNE-1 and 2mHNE-2 (Table 1 and Fig. S2†). We then subjected the enriched library to an additional five rounds of selection in which the stringency was further increased by increasing the ionic strength of the wash buffer (by adding up to 500 mM NaCl) and by adding increasing amounts of fetal bovine serum (FBS; up to 80%; see Table S1†). Sequencing 30 library members revealed four additional sequences that were enriched, 2mHNE-3–2mHNE-6 (Table 1).

**Table 1:** Sequences of selected 2′-OMe aptamers and their affinities for HNE

Aptamer	Random region sequence	*K*_d_ (nM)
2mHNE-1	CCCTGTTCCCCCTCCGCCGCTTGACTCTCC	520 ± 50
2mHNE-2	CCCTGGCCGCCCTCTGGCGCGCTGATGT	180 ± 10
2mHNE-3	GCCCTACATTCTGTGCGACTCCCCCCCCCGC	120 ± 7
2mHNE-4	CCCTTACCTACTCTGCTCCCCCCCCCCCTCGGCTGC	45 ± 4
2mHNE-5	CCCTGTTCGCTATCCCCCATCCCCCGATTGC	110 ± 20
2mHNE-6	CCCGCTGTTCTCCCCCCCCCGCTGAGATCCCTGT	110 ± 20

The identified oligonucleotides were individually prepared *via* transcription with a fully 2′-OMe-modified primer containing a 5′-Alexa 488 fluorophore and their affinity for HNE was characterized using microscale thermophoresis (MST) with binding buffer supplemented with 0.05% Triton X-100 and 0.1% PEG 8000 (Table 1 and Fig. S3†). The round-five aptamers 2mHNE-1 and 2mHNE-2 bound to HNE with *K*_d_ values of 520 and 180 nM. The round-ten aptamers bound with higher affinity, with *K*_d_ values ranging from 45 nM to 120 nM. For comparison, we prepared and analyzed the previously reported, fully natural HNE aptamer DNA-I,^18^ which under identical conditions bound to HNE with a *K*_d_ of 280 nM.

To determine whether the 2′-OMe substituents contributed to function, we prepared the fully natural counterparts (HNE-1–HNE-6) by PCR amplification of chemically synthesized DNA templates, with one primer that was labeled with a 5′-Alexa 488 fluorophore and one that was labeled with a 5′-biotin. The PCR product was immobilized on streptavidin beads, which were then washed with NaOH to elute the desired ssDNA aptamers. *K*_d_ values for HNE-1–HNE-6 were determined by MST to be 500, 520, 300, 340, 480, and 550 nM, respectively (Fig. S3†). Thus, with the exception of the round 5 aptamer 2mHNE-1, the 2′-OMe substituents contribute significantly to HNE binding.

**Fig. 2:**
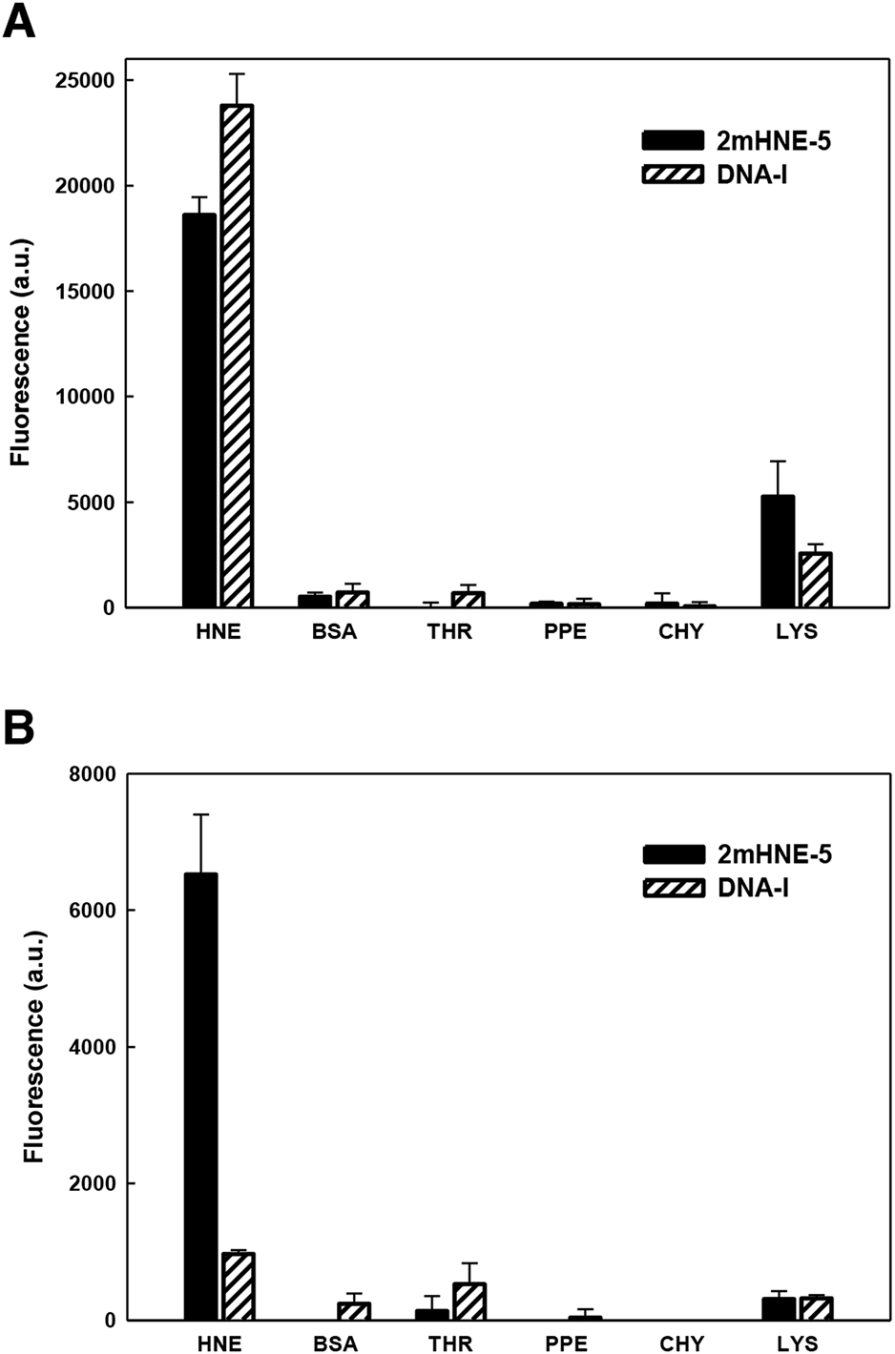
Specificity of binding of 2mHNE-5 and DNA-I to different proteins in the presence of 150 mM NaCl (A) or 500 mM NaCl, 66 μg mL^–1^ yeast tRNA (B). BSA, bovine serum albumin; THR, thrombin; PPE, porcine pancreas elastase; CHY, chymotrypsin; LYS, lysozyme. Data are the average and s.d. of three independent determinations.

As mentioned above, aptamers often bind positively charged proteins, such as HNE (pI ≈ 9), *via* non-specific electrostatic interactions, raising specificity as a concern. Indeed, previous studies revealed that DNA-I does not retain affinity for HNE in the presence of high salt concentrations.^15^ We thus examined the specificity with which 2mHNE-5 binds to HNE compared to other proteins with various pI values, under both low (150 mM) and high (500 mM) salt conditions, using a fluorescence-based plate assay. Briefly, HNE, bovine serum albumin (pI ≈ 4.7), thrombin (pI ≈ 7), porcine pancreas elastase (pI ≈ 8.5), chymotrypsin (pI ≈ 8.8), or lysozyme (pI ≈ 11) was non-specifically immobilized to the surface of individual wells of 96-well microtiter plates; 2mHNE-5 or DNA-I, both 5′-Alexa 488 labeled, was added to a final concentration of 10 nM; and after the plates were washed with binding buffer (150 mM NaCl) or high salt binding buffer (500 mM NaCl and 66 μg mL^–1^ yeast tRNA), fluorescence was measured (Fig. 2). Under low salt conditions, despite the concentration of the aptamers being significantly below the *K*_d_ values measured by MST, both 2mHNE-5 and DNA-I bound to HNE, but did not bind strongly to any of the other proteins (although both aptamers exhibited some affinity for lysozyme). However, under the high salt conditions, 2mHNE-5 specifically bound HNE while DNA-I did not, demonstrating that the 2′-OMe modified aptamer binds to HNE *via* more specific interactions.

**Fig. 3:**
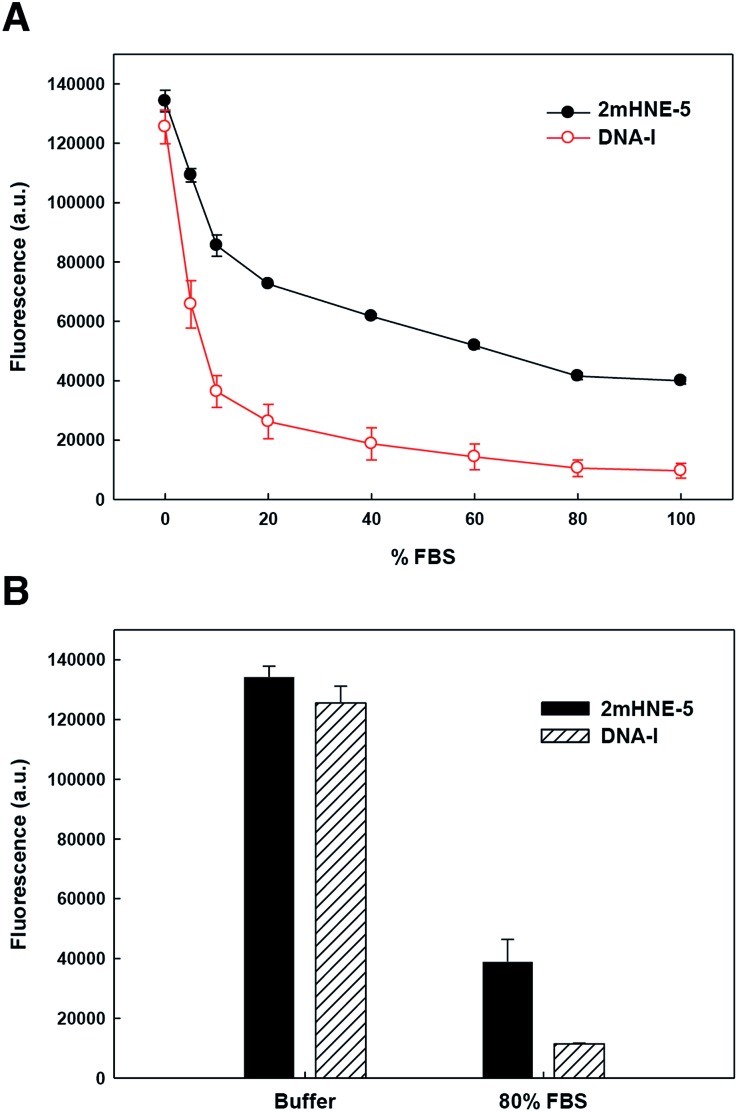
Binding of 2mHNE-5 and DNA-I to HNE in the presence of increasing concentrations of FBS in the wash buffer (A) or in the presence of 80% FBS (B). Data are the average and s.d. of three independent determinations.

To explore the ability of the aptamers to bind HNE in serum, we repeated the above described low-salt plate binding assays with 2mHNE-5 or DNA-I, but with increasing amounts of FBS in the wash buffer (Fig. 3A). The results clearly show that DNA-I rapidly loses affinity for HNE as the percentage of FBS is increased. In contrast, 2mHNE-5 loses affinity more slowly and even retains the ability to bind HNE during washing with 100% FBS. We also directly measured binding in the presence of 80% FBS, and found that 2mHNE-5 clearly binds HNE better than does the DNA-I control (Fig. 3B).

**Fig. 4:**
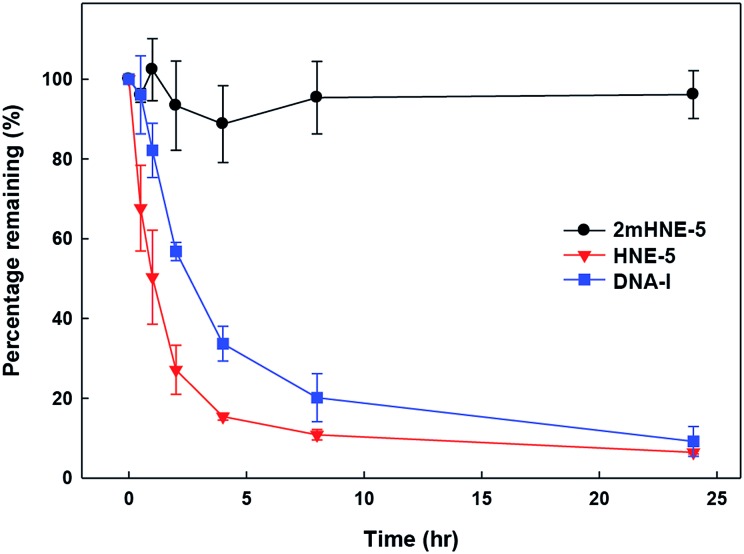
Stability of 2mHNE-5, HNE-5, and DNA-I in FBS. Data are the average and s.d. of three independent determinations.

To confirm that the 2′-OMe substituents do confer 2mHNE-5 with resistance to nuclease degradation, folded 2mHNE-5, HNE-5, or DNA-I was mixed with undiluted FBS (Fig. 4). After incubation at 37 °C, PAGE analysis revealed the degradation of DNA-I, with little aptamer remaining after 4 h. In contrast, even after 24 h, no degradation of 2mHNE-5 was observed, clearly demonstrating that the 2′-OMe modifications do indeed provide significant stabilization against nuclease degradation. HNE-5 was degraded even faster than DNA-I, likely due to the absence of secondary structure, suggesting that the 2′-substituents of 2mHNE-5 are required for proper folding.

## Conclusions

There has been both academic and industrial interest in developing 2′-OMe aptamers,^4^,^11^,^19^ but their identification has historically been challenging, because aptamer selections rely on polymerase recognition, and the modified triphosphates are not well recognized. In fact, to our knowledge, prior to this work no selections have been performed in which the modified libraries were produced using only 2′-OMe triphosphates. The directed evolution of SFM4-6 and SFM4-9 now allows for the “transcription” and “reverse transcription” of fully modified aptamers using only 2′-OMe modified triphosphates and we have used them here to select 2′-OMe modified aptamers that bind HNE. Importantly, the OMe substituents of the selected aptamers are required for high affinity and specific HNE binding. Likely, both the affinity and specificity afforded by the modifications could be increased further by additional diversification and selection, including negative selection against binding related proteins. Finally, the availability of SFM4-6 and SFM4-9 should enable the facile selection of other 2′-OMe aptamers and the exploration of the effects of the added OMe groups beyond imparting the aptamers with resistance to nucleases.

## Conflicts of interest

There are no conflicts to declare.

## Supplementary Material

Supplementary information
