# Supplementary material for: Evolved polymerases facilitate selection of fully 2′-OMe-modified aptamers
Source: Chem Sci. 2017 Oct 16;8(12):8179–82. doi: 10.1039/c7sc03747c (PMC5855981; doi:10.1039/c7sc03747c)
Supplement: Supplementary file 1 [file SC-008-C7SC03747C-s001.pdf]

Electronic Supplementary Material (ESI) for Chemical Science.
This journal is © The Royal Society of Chemistry 2017

Electronic Supplementary Information

Evolved polymerases facilitate selection of fully 2′-OMe-modified aptamers

Zhixia Liu, Tingjian Chen, and Floyd E. Romesberg
Department of Chemistry, The Scripps Research Institute, La Jolla, CA 92037 USA

Methods

2′-OMe-ssDNA library preparation

The fully 2′-OMe modified ssDNA library was prepared by "transcription" using a previously evolved Stoffel fragment DNA polymerase SFM4-6.[1] Fully 2′-OMe-modified primer mP-lower (1 μM) was added to the DNA library template T6-lib (0.5 μM) and annealed by heating to 95 °C for 5 min and cooling slowly to 10 °C. A final concentration of 1 μM of SFM4-6 was added along with a reaction buffer containing 1× standard Taq buffer (New England Biolabs (NEB), USA) and 1× OneTaq buffer (NEB, USA) supplemented with 4.7 mM $MgCl_2$, 1 mM $MnCl_2$, and 0.5 mM each of all four 2′-OMe-NTPs (TriLink BioTechnologies, USA). The reaction mixture was incubated at 50 °C for 2 h, followed by incubation at 70 °C for 20 min. This temperature cycle was repeated 5 additional times, after which the reaction was incubated at 70 °C for 1 h to finish synthesis. After transcription, TURBO DNase (Thermo Fisher Scientific, USA) was added and the reaction was incubated at 37 °C for at least 2 h to digest the DNA template. Next, the mixture was column purified using ssDNA/RNA Clean & Concentrator (Zymo Research, USA) to generate a 2′-OMe-ssDNA library for the first round of SELEX. 2′-OMe DNA primers and any truncated transcription products that are not digested by DNase or removed by column purification were also subjected to selection as additional competitors, as these library members cannot be recovered after selection because they lack the hybridization region for the reverse transcription primer.

In vitro selection

One well of a 96-well ELISA plate (Thermo Fisher Scientific, USA) was coated with HNE (600 nM) (Innovative Research, USA) in binding buffer (20 mM HEPES pH 7.5, 150 mM NaCl, 6 mM KCl, 2 mM $MgCl_2$) overnight at 4 °C, then washed 5 times with binding buffer. The 2′-OMe-ssDNA library was first folded in binding buffer by heating to 95 °C for 5 min and cooling slowly to room temperature, then added to the HNE-coated well, and incubated for 1 h at room temperature. In later rounds when yeast-tRNA or fetal bovine serum (FBS, Sigma-Aldrich, USA) was included during binding as a non-specific binding competitor or complex media, it was added to the library after folding but before adding the library to the well. In the case of rounds with a negative selection step, the library was first incubated in an empty well or a BSA-coated well for 1 h before being transferred to the HNE coated well. After incubation the unbound library was removed, and the well was washed 6 times with buffer or FBS as indicated in Table S2. The bound aptamers were eluted with formamide pre-heated to 95 °C, and the eluate was column purified using ssDNA/RNA Clean & Concentrator and reverse transcribed into DNA for PCR amplification. All fractions including unbound, washes and eluate were collected, reversed transcribed and subjected to qPCR analysis to monitor the enrichment from different rounds.

Reverse transcription and PCR amplification

"Reverse transcription" of 2′-OMe-DNA back into DNA was carried out with the evolved DNA polymerase SFM4-9.[1] The eluate was annealed to a DNA primer with a poly $d(T)_{20}$ tail and C18 spacer, Sp18-P-upper, and mixed with SFM4-9 (2.5 μM) in a reaction buffer containing 1× standard Taq buffer supplemented with 1 mM of dNTPs, 1 mM of $MnCl_2$, 2 mM of $MgCl_2$, 0.1% of Triton X-100, and 0.01% of BSA. The reaction was incubated at 50 °C for 12 h, followed by incubation at 72 °C for 2 h to finish synthesis. The mixture was then subjected to PCR amplification.

A small percentage (2%) of the reverse transcription mixture was added as template in a PCR reaction and amplified with OneTaq DNA Polymerase (NEB, USA) and DNA primers Sp18-P-upper and P-lower, following the protocol provided by NEB. (Poly d(T)$_{20}$ was introduced for the ease of following gel separation by size. The Sp18 spacer was introduced to prevent the transcription of poly d(T)$_{20}$.) The thermocycling program used was: 94 ° C for 30 s, *n* cycles of (94 °C for 20 s; 51.2°C for 40 s; 68 °C for 10 s), 68 °C for 5 min. The number of cycles (*n*) was optimized for each selection round by running a pilot 25-cycle qPCR to prevent over amplification of the library, typical values of *n* were 15–18. The amplified DNA was loaded on a 15% denaturing polyacrylamide gel containing 8 M urea to separate the two strands by size. The longer strand containing the poly d(T)$_{20}$ tail and C18 spacer was recovered to be used as ssDNA template to "transcribe" 2′-OMe-ssDNA library for the next selection round as described above. For sequencing, libraries were cloned into pET-23b, transformed into *E. coli* DH5α and sequenced to identify individual sequences from the libraries.

Preparation of 2′-OMe aptamers

Aptamer sequences of interest were chosen based on sequencing results, and their complementary DNA templates (HNE-Tp-1 to HNE-Tp-6) were synthesized by Integrated DNA Technologies (IDT, USA). The 2′-OMe aptamers were synthesized from these DNA templates and 5′-Alexa 488 labeled 2′-OMe primer A488-mP-lower following the transcription procedure described above. The DNA template was digested with TURBO DNase, and the 5′-Alexa 488 labeled 2′-OMe aptamer was purified and recovered from a 15% denaturing polyacrylamide gel. Aptamer concentration was determined by fluorescence (λ_{ex} = 485 nm; λ_{em} = 535 nm) using an Envision Multilabel Plate Reader (Perkin Elmer) and a standard curve that was constructed using known concentrations of 5′-Alexa 488 labeled primer.

Preparation of natural DNA aptamers

Natural DNA aptamers were prepared by PCR amplification with OneTaq DNA Polymerase using corresponding DNA templates (HNE-Tp-1 to HNE-Tp-6), biotinylated DNA primer Biotin-P-upper and 5′-Alexa 488 labeled DNA primer A488-P-lower following standard protocols. The resulting PCR product was column purified with DNA Clean & Concentrator and then mixed with streptavidin-coated beads (Dynabeads MyOne Streptavidin C1, Thermo Fisher Scientific, USA). The 5′-Alexa 488-labeled DNA aptamer strand was generated by eluting from the beads with 0.1 M NaOH and purifying using ssDNA/RNA Clean & Concentrator. Aptamer concentration was determined using the method described above.

Determination of binding affinity

The binding affinity of 2′-OMe aptamers to HNE was characterized by microscale thermophoresis (MST) using a Monolith NT.115 instrument (NanoTemper Technologies). Aptamer was kept at a constant concentration of 20 nM; HNE concentration was varied. Alexa 488-labeled 2′-OMe or DNA aptamer was folded and then incubated with HNE for 20 min at room temperature in binding buffer with an additional 0.05% Triton X-100 and 0.1% PEG 8000. The solution was briefly centrifuged and then loaded in NanoTemper premium capillaries. A laser power of 50% and MST power of 80% was used for the assay. All experiments were repeated in triplicate. The data was analyzed using NanoTemper analysis software to obtain the dissociation constant K_d, where [B_0] is total concentration of binding sites, [L_0] is total ligand concentration, [BL] is the concentration of bound complex. Knowing [B_0], [L_0] and [BL], the value of K_d was calculated:[2]

$$\frac{[\mathrm{BL}]}{[B_0]} = \frac{([L_0] + [B_0] + K_d) - \sqrt{(([L_0] + [B_0] + K_d))^2 - 4[L_0][B_0]}}{2[B_0]}$$

Our data was best fitted using the Hill equation, which is suitable for reactions with more than one ligand binding to one binding partner or for reactions in which there is cooperativity upon binding:

$$\frac{[\mathrm{BL}]}{[B_0]} = \frac{1}{1 + K_d/([L])^h}$$

where h is the Hill coefficient, K_d is the dissociation constant and $[L]$ is the concentration of ligand.[2]

Binding specificity assay

Wells of a black 96-well ELISA plate were coated with 560 ng of HNE (pI ≈ 9), BSA (pI ≈ 4.7) (NEB, USA), human alpha thrombin (pI ≈ 7) (Haematologic Technologies, USA), porcine pancreas elastase (pI ≈ 8.5) (Sigma-Aldrich, USA), human pancreas chymotrypsin (pI ≈ 8.8) (Athens Research & Technology, USA) or lysozyme (pI ≈ 11, Sigma-Aldrich, USA) overnight at 4 °C, then washed 5 times with binding buffer. 10 nM of 5′-Alexa 488 labeled aptamers were folded as described above, added to each well, and incubated at room temperature for 1 h. For binding in the presence of yeast tRNA, yeast tRNA was added to folded aptamers to a final concentration of 66 μg/ml. The wells were washed 5 times with binding buffer and fluorescence was recorded as described above. For the high-salt condition, high salt binding buffer (20 mM HEPES, pH 7.5, 500 mM NaCl, 6 mM KCl, 2mM $MgCl_2$) was used for washing. All experiments were repeated in triplicate.

Determination of the effect of serum on aptamer binding to HNE

For the FBS washing experiment, black 96-well ELISA plate wells were coated with HNE in binding buffer (20 mM HEPES, pH 7.5, 150 mM NaCl, 6 mM KCl, 2 mM $MgCl_2$) overnight at 4 °C, then washed 5 times with binding buffer. 5′-Alexa 488 labeled aptamers were folded first at 300 nM in binding buffer by heating to 95 °C for 5 min and cooling slowly to room temperature, then incubated in the HNE-coated wells for 1 h at room temperature. The wells were washed with binding buffer 3 times and fluorescence was recorded as described above. Next, the wells were washed with 5% FBS by incubating for 10 min at room temperature followed by washing 3 times with binding buffer, and fluorescence was recorded. Using this approach, the FBS concentrations used for washing were gradually increased up to 100% and corresponding signals were recorded.

For the FBS binding experiment, the plate wells were coated with HNE in same way described above. 5′-Alexa 488 labeled aptamers (1500 nM) were first folded in binding buffer by heating to 95 °C for 5 min and cooling slowly to room temperature, then they were mixed with undiluted serum to a final aptamer concentration of 300 nM (FBS = 80%). The mixtures were added to the HNE-coated wells and incubated for 1 h at room temperature. The wells were washed with binding buffer for 3 times and fluorescence was recorded as described above. All experiments were repeated in triplicate.

Stability in serum

5′-Alexa 488 labeled aptamers (500 nM) were first folded in binding buffer by heating to 95 °C for 5 min and cooling slowly to room temperature, then mixed with undiluted FBS (final FBS = 90%). The mixture were immediately incubated at 37 °C. Aliquots were taken at 0, 0.5, 1, 2, 4, 8 and 24 hr. Formamide gel loading buffer and an additional 20 mM of EDTA were added immediately to the aliquots to quench degradation. All samples were kept at -20 °C until analysis on 20% denaturing polyacrylamide gels containing 8 M urea. All experiments were repeated in triplicate.

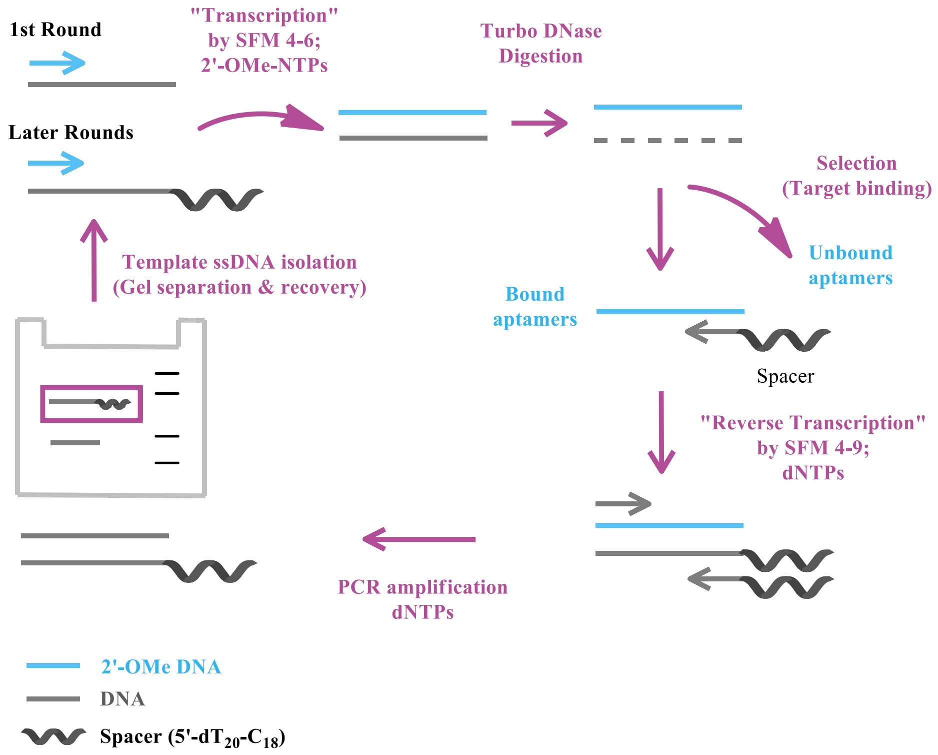

Scheme S1. 2′-OMe-DNA library preparation and selection

Table S1. Selection pressure used in SELEX

Round	Negative Selection (well surface)	Negative Selection (BSA)	Wash Steps	Wash Ionic Strength	Competitor (yeast tRNA)	Complex Media for binding (FBS)
**1**	No	No	6× 2 min	150 mM		
**2**	Yes	Yes	6× 2 min	150 mM	0.015 μg/μL	
**3**	Yes	Yes	6× 2 min	150 mM	0.075 μg/μL	
**4**	Yes	Yes	6× 2 min	150 mM	0.5 μg/μL	
**5**	Yes	Yes	6× 10 min	150 mM	3 μg/μL	
**6**	Yes	Yes	6× 10 min	500 mM	3 μg/μL	
**7**	Yes	Yes	(1) 10% FBS, 10 min<br>(2) 6× 10 min	500 mM	3 μg/μL	10%
**8**	Yes	Yes	(1) 10% FBS, 10 min<br>(2) 6× 10 min	500 mM	3 μg/μL	10%
**9**	Yes	Yes	(1) 100% FBS, 10 min<br>(2) 6× 10 min	500 mM	3 μg/μL	80%
**10**	Yes	Yes	(1) 100% FBS, 10 min<br>(2) 6× 10 min	500 mM	3 μg/μL	80%

Table S2. DNA sequences (m: 2′-OMe-modified nucleotide)

Name	Sequence
T6-lib	5′-GTAGTCAGTAGTCTAGCANNNNNNNNNNNNNNNNNNNNNNNNNNNNNNCCCTAATAGTGAGTCGTAT
P-lower	5′-ATACGACTCACTATTAGGG
mP-lower	5′-mAmUmAmCmGmAmCmUmCmAmCmUmAmUmUmAmGmGmG
A488-mP-lower	/5Alex488N/mAmUmAmCmGmAmCmUmCmAmCmUmAmUmUmAmGmGmG
A488-P-lower	/5Alex488N/ ATACGACTCACTATTAGGG
Sp18-P-upper	5′-TTTTTTTTTTTTTTTTTTTT/iSp18/GTAGTCAGTAGTCTAGCA
Biotin-P-upper	/5BiotinTEG/GTAGTCAGTAGTCTAGCA
HNE-Tp-1	5'-GTAGTCAGTAGTCTAGCAGGAGAGTCAAGCGGCGGAGGGGGAACAGGGCCCTAATAGTGAGTCGTAT
HNE-Tp-2	5'-GTAGTCAGTAGTCTAGCAACATCAGCGCGCCAGAGGGCGGCCAGGGCCCTAATAGTGAGTCGTAT
HNE-Tp-3	5'-GTAGTCAGTAGTCTAGCAGCGGGGGGGGAGTCGCACAGAATGTAGGGCCCCTAATAGTGAGTCGTAT
HNE-Tp-4	5'-GTAGTCAGTAGTCTAGCAGCAGCCGAGGGGGGGGGGGAGCAGAGTAGGTAAGGGCCCTAATAGTGAGTCGTAT
HNE-Tp-5	5'-GTAGTCAGTAGTCTAGCAGCAATCGGGGGATGGGGGATAGCGAACAGGGCCCTAATAGTGAGTCGTAT
HNE-Tp-6	5'-GTAGTCAGTAGTCTAGCAACAGGGATCTCAGCGGGGGGGGGAGAACAGCGGGCCCTAATAGTGAGTCGTAT
A488-DNA-I	/5Alex488N/TAGCGATACTGCGTGGGTTGGGGCGGGTAGGGCCAGCAGTCTCGT

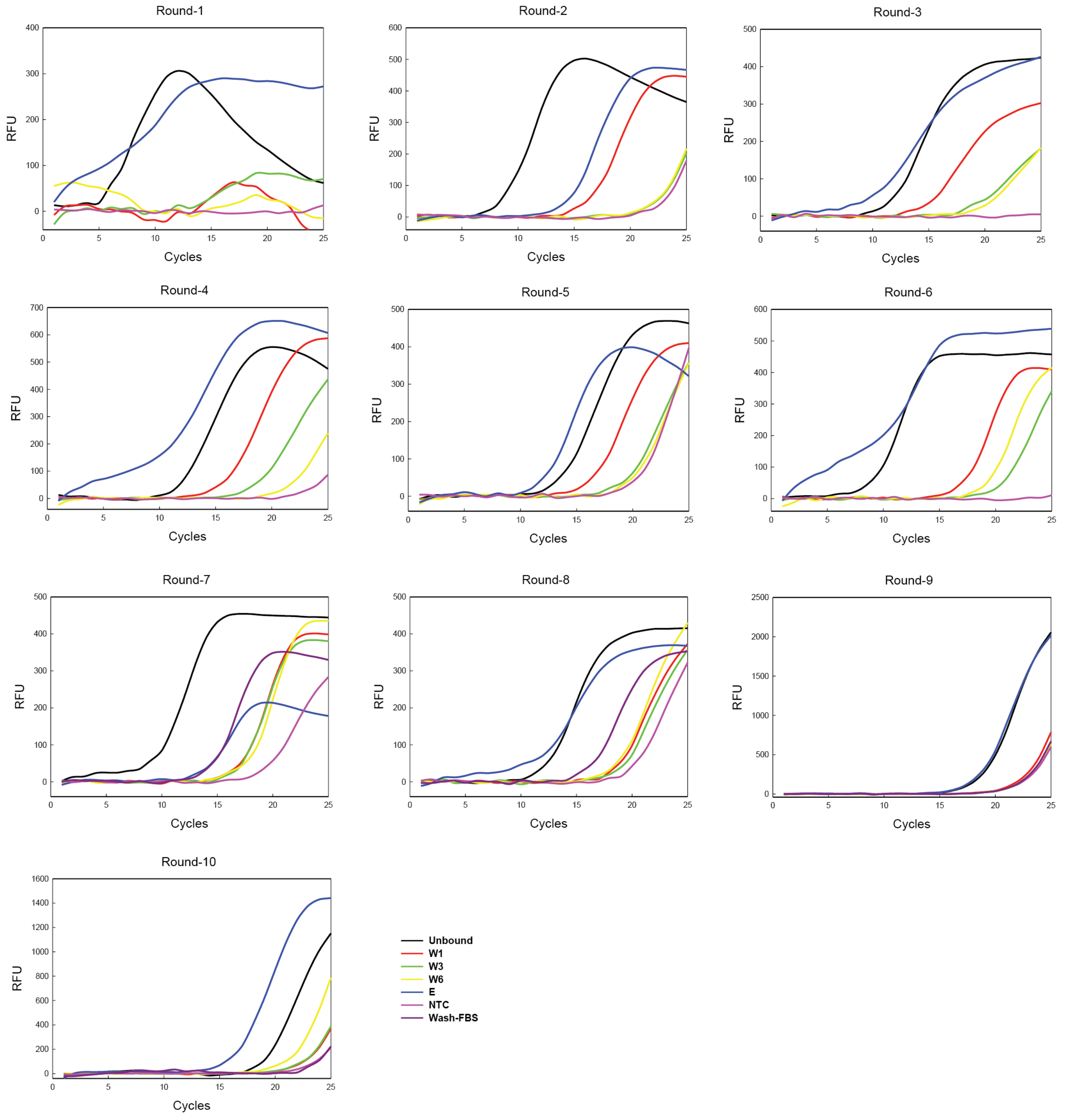

Figure S1. qPCR curves of selection fractions from each round for amplification cycle optimization and monitoring eluate enrichment (only the fractions Unbound, Wash 1, Wash 3, Wash 6, Wash by FBS, Eluate and non-template control (NTC) are shown).

	5'-constant region	random region	3'-constant region
**Round 5**			
2mHNE-1	*mAmUmAmCmGmAmCmUmCmAmCmUmAmUmUmAmGmGmG*	**mCmCmCmUmGmUmUmCmCmCmCmCmUmCmCmGmCmCmGmCmUmUmGmAmCmUmCmUmCmC**	*mUmGmCmUmAmGmAmCmUmAmCmUmGmAmCmUmAmC*
2mHNE-2	*mAmUmAmCmGmAmCmUmCmAmCmUmAmUmUmAmGmGmG*	**mCmCmCmUmGmGmCmCmGmCmCmCmUmCmUmGmGmCmGmCmGmCmUmGmAmUmGmU**	*mUmGmCmUmAmGmAmCmUmAmCmUmGmAmCmUmAmC*
**Round 10**			
2mHNE-3	*mAmUmAmCmGmAmCmUmCmAmCmUmAmUmUmAmGmGmG*	**mGmCmCmCmUmAmCmAmUmUmCmUmGmUmGmCmGmAmCmUmCmCmCmCmCmCmCmCmGmC**	*mUmGmCmUmAmGmAmCmUmAmCmUmGmAmCmUmAmC*
2mHNE-4	*mAmUmAmCmGmAmCmUmCmAmCmUmAmUmUmAmGmGmG*	**mCmCmCmUmUmAmCmCmUmAmCmUmCmUmGmCmUmCmCmCmCmCmCmCmCmCmCmCmUmCmGmGmCmUmGmC**	*mUmGmCmUmAmGmAmCmUmAmCmUmGmAmCmUmAmC*
2mHNE-5	*mAmUmAmCmGmAmCmUmCmAmCmUmAmUmUmAmGmGmG*	**mCmCmCmUmGmUmUmCmGmCmUmAmUmCmCmCmCmCmAmUmCmCmCmCmCmGmAmUmUmGmC**	*mUmGmCmUmAmGmAmCmUmAmCmUmGmAmCmUmAmC*
2mHNE-6	*mAmUmAmCmGmAmCmUmCmAmCmUmAmUmUmAmGmGmG*	**mCmCmCmGmCmUmGmUmUmCmUmCmCmCmCmCmCmCmCmCmGmCmUmGmAmGmAmUmCmCmCmUmGmU**	*mUmGmCmUmAmGmAmCmUmAmCmUmGmAmCmUmAmC*

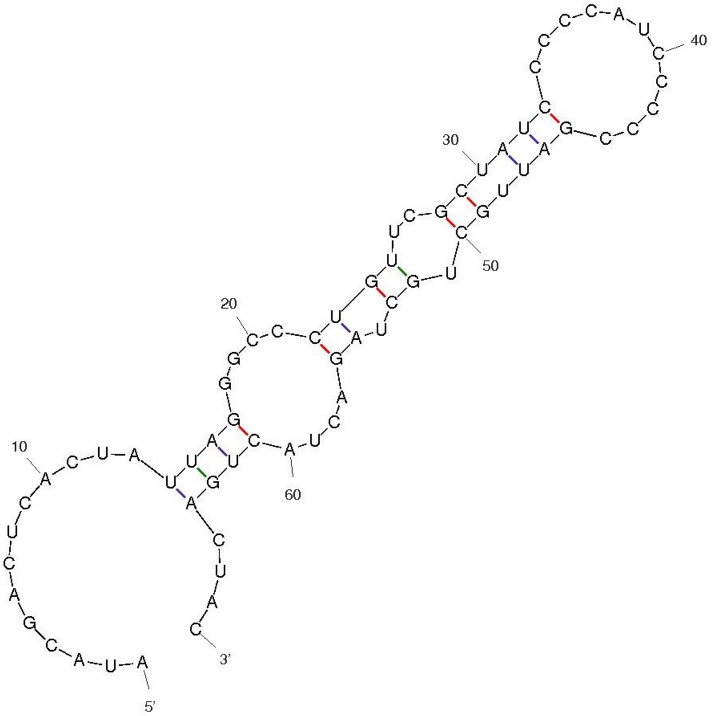

Figure S2. Aptamer sequences (m: 2′-OMe-modified nucleotide) and representative secondary structure (2mHNE-5) predicted using the program mFold.[3]

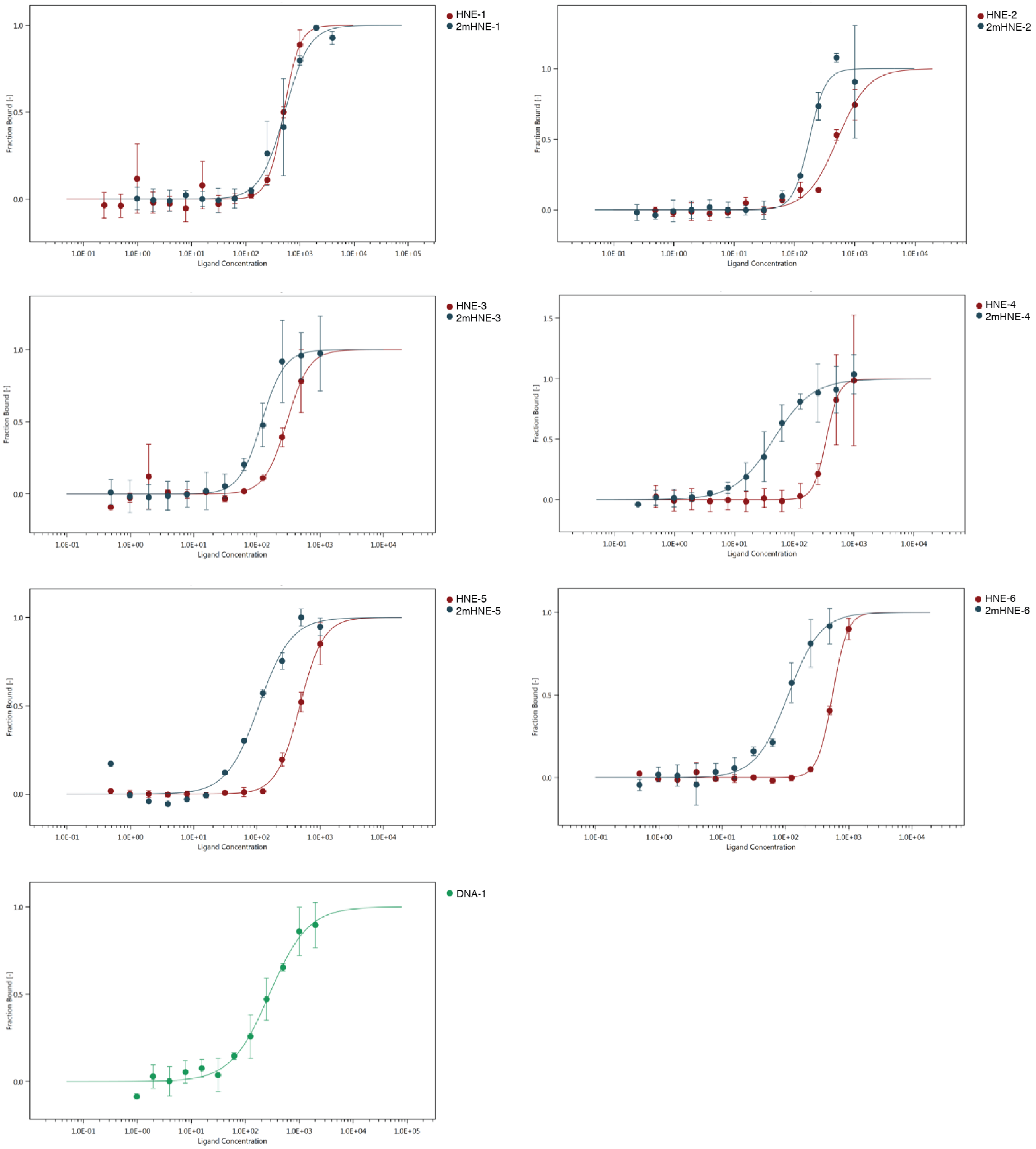

Figure S3. MST data fitting curves

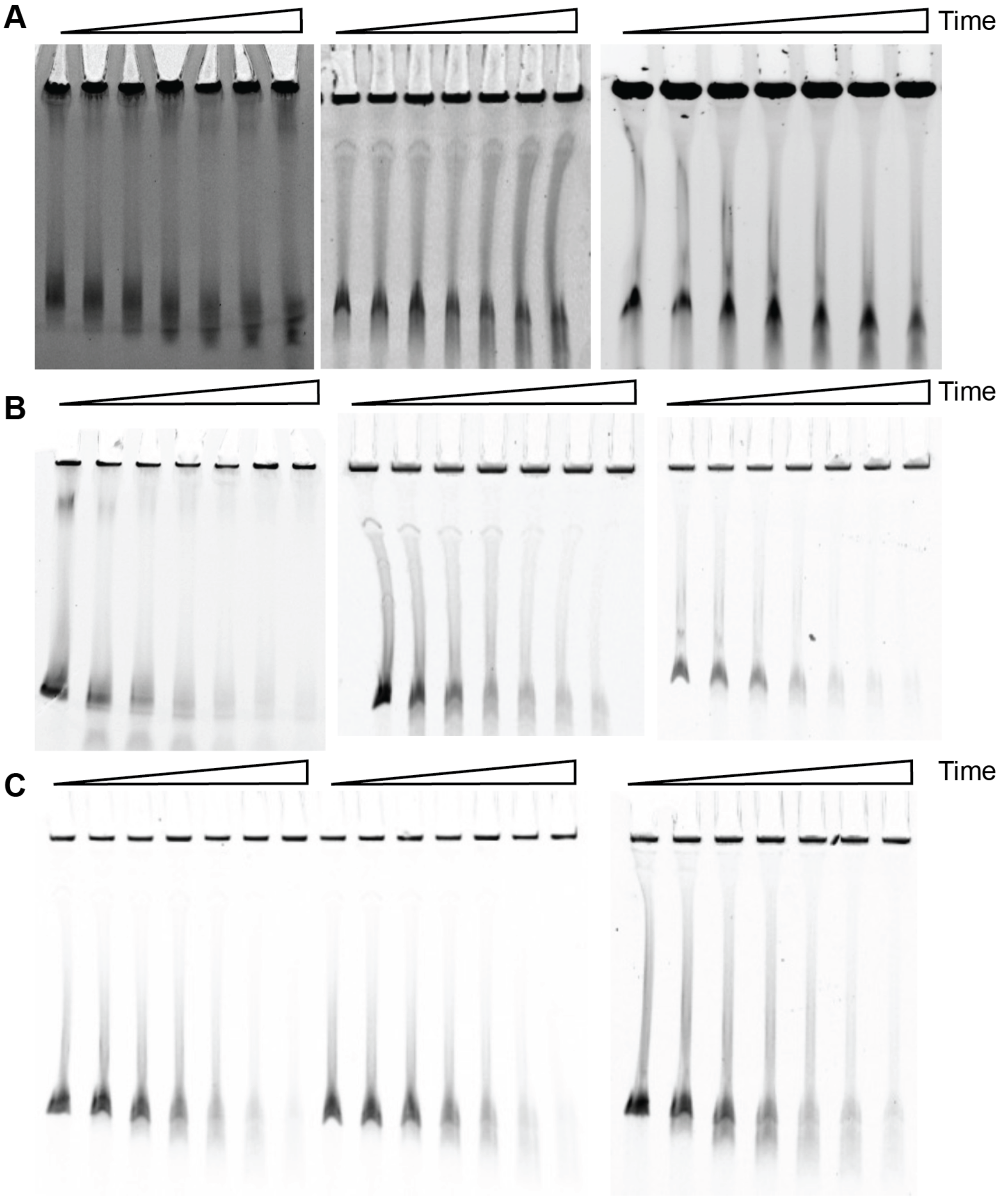

Figure S4. Gels for aptamer stability in serum (A. 2mHNE-5; B. HNE-5; C. DNA-I at time points of 0, 0.5, 1, 2, 4, 8 and 24 hr)

References

1. T. Chen et al. *Nat. Chem.*, 2016, **8**, 556–562.
2. C. J. Wienken, P. Baaske, U. Rothbauer, D. Braun & S. Duhr. *Nat. Commun.*, 2010, **1**, 100.
3. M. Zuker. *Nucleic Acids Res.*, 2003, **31**, 3406–3415.
